# The Use of I.V. Albumin During Kidney Replacement Therapy: A Survey of Nephrologists and Intensivists

**DOI:** 10.1016/j.ekir.2021.11.031

**Published:** 2021-12-08

**Authors:** Ryan J. Chan, William Beaubien-Souligny, Samuel A. Silver, Sean M. Bagshaw, Ron Wald, Pierre-Antoine Brown, Swapnil Hiremath, Jennifer W.Y. Kong, Edward G. Clark

**Affiliations:** 1 Department of Medicine, University of Ottawa, Ottawa, Ontario, Canada; 2 Division of Nephrology, Department of Medicine, Centre Hospitalier de l’Université de Montréal, Montréal, Quebec, Canada; 3 Division of Nephrology, Department of Medicine, Queen’s University, Kingston, Ontario, Canada; 4 Department of Critical Care Medicine, Faculty of Medicine and Dentistry, University of Alberta, Edmonton, Alberta, Canada; 5 Division of Nephrology, St. Michael’s Hospital (Unity Health), University of Toronto, Toronto, Ontario, Canada; 6 Division of Nephrology, Department of Medicine, The Ottawa Hospital, University of Ottawa, Ottawa, Ontario, Canada; 7 Kidney Research Centre and Clinical Epidemiology Program, Ottawa Hospital Research Institute, Ottawa, Ontario, Canada

## Introduction

Hemodynamic instability during kidney replacement therapy (KRT) occurs frequently with KRT modalities used in the intensive care unit (ICU), including intermittent hemodialysis, slow low-efficiency dialysis, and continuous KRT.^1^ In critically ill patients, hemodynamic instability during KRT is associated with an increased risk of death.^2^ The STARRT-AKI (Standard vs. Accelerated Initiation of Renal Replacement Therapy in Acute Kidney Injury trial)^3^ found that earlier initiation of KRT for AKI was associated with more frequent hemodynamic instability and less recovery to KRT independence. This aligns with other indirect evidence^4^ that hemodynamic instability during KRT might decrease the likelihood of kidney recovery after AKI. Nevertheless, i.v. hyperoncotic albumin may prevent hemodynamic instability and facilitate targeted ultrafiltration in critically ill patients on KRT,^5^^,^^6^ but it is very costly and has a weak evidence base.^7^ Despite this, there is some evidence that i.v. albumin is already frequently prescribed for this indication.^7^^,^^8^

To better inform the design of future clinical trials, we undertook a survey to describe current practices and attitudes regarding the use of i.v. albumin based on hemodynamics and other clinical parameters in critically ill patients on KRT. This was done through a survey of the physician membership of both the Canadian Society of Nephrology and the Canadian Critical Care Society. The survey questions (including complete reporting of survey responses) and description of data collection and analysis are detailed in the Supplementary Methods.

**Figure 1:**
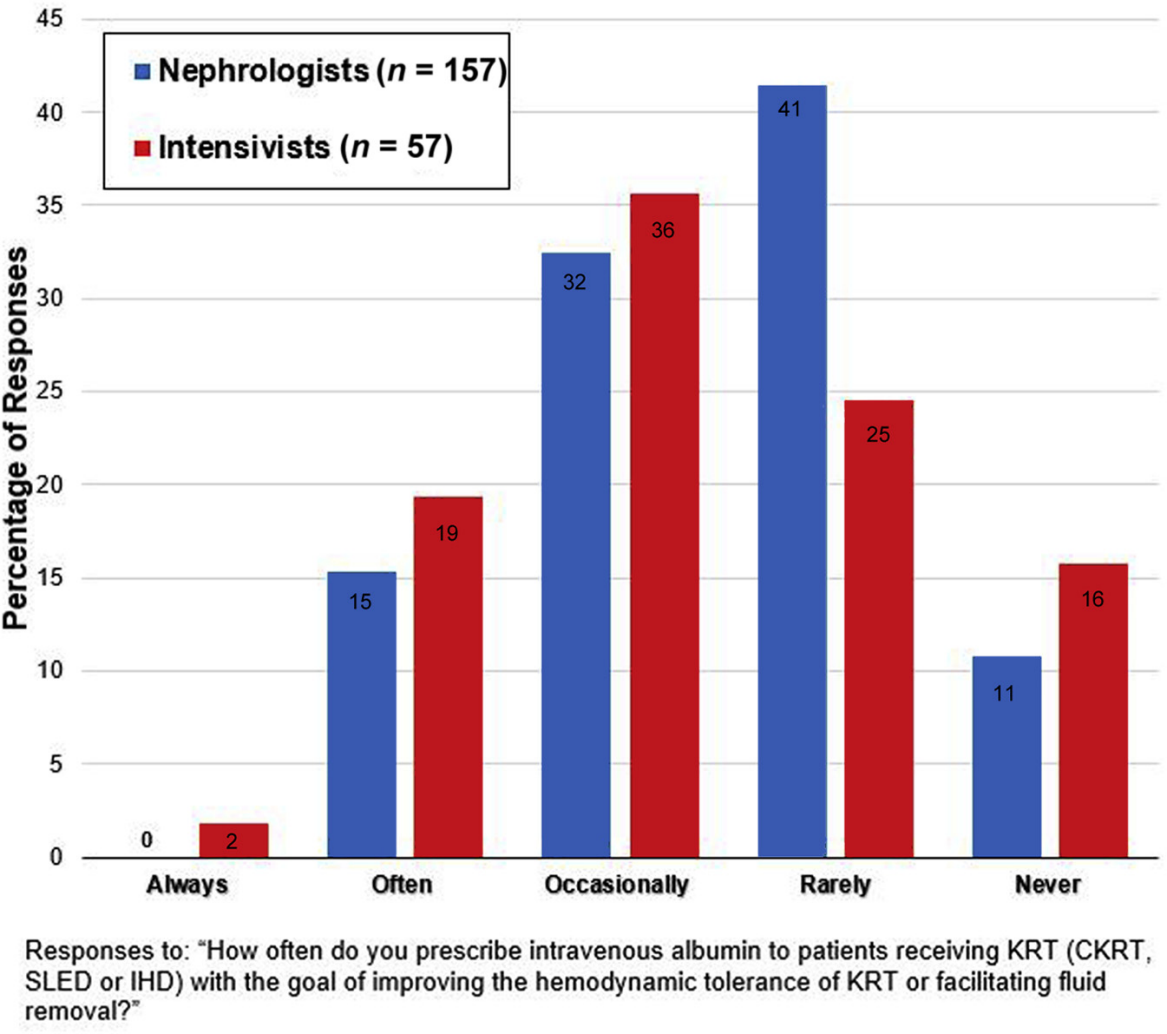
Self-reported i.v. albumin-prescribing practices for patients on KRT in the IHD. CKRT, CKRT, continuous kidney replacement therapy; IHD, intensive care unit; KRT, kidney replacement therapy; SLED, slow low-efficiency dialysis.

Of 268 Canadian Society of Nephrology physician members and 324 members of the Canadian Critical Care Society (including non-physicians), 162 nephrologists (60%) and 59 intensivists (18%) involved in providing KRT in the ICU setting responded to the survey, respectively. Continuous KRT was the modality most often used in hemodynamically unstable patients at respondents’ primary practice locations (69% of nephrologists, 79% of intensivists), followed by slow low-efficiency dialysis (26% and 16%, respectively) and intermittent hemodialysis (5% of both groups). Figure 1 reports the frequency with which respondents indicated they prescribe albumin to patients receiving KRT in ICU with the goal of “improv[ing] hemodynamic tolerance or facilitat[ing] fluid removal”; overall, intensivists were significantly more likely than nephrologists to prescribe albumin “occasionally” or more (60% vs. 48%, respectively) for this indication (*P* = 0.02). Nevertheless, there was no significant difference between groups in establishing a threshold serum albumin concentration at which i.v. albumin would always or never be prescribed.

Table 1 details responses to a clinical scenario revealing a variety of parameters influence the likelihood that albumin is prescribed. We described a case in which a 75-year-old woman was admitted to the ICU with pneumonia and fluid overload and required a moderate dose of vasopressors owing to hypotension. Her serum albumin level was 25 g/l, she had severe AKI, and she was started on KRT targeting fluid removal to improve her respiratory status. Presented with this initial scenario, 22% of nephrologists and 23% of intensivists indicated that they were “likely” or “very likely” to prescribe or suggest the administration of albumin during KRT to enhance hemodynamic stability or facilitate fluid removal. As detailed in Table 1, additional questions evaluated the likelihood that nephrologists and intensivists would prescribe or suggest the use of albumin according to the provision of additional information, such as whether ultrafiltration was limited during the last KRT session owing to hemodynamic instability or whether KRT was being provided before extubation. Taken together, the survey respondents (intensivists and nephrologists) were significantly more likely to prescribe albumin when told that the KRT modality was intermittent hemodialysis as opposed to continuous KRT (62% vs. 3%, respectively). They were also significantly more likely to give albumin when the patient was on vasopressors as opposed to not on vasopressors (60% vs. 7%, respectively) or when the serum albumin level was <20 g/l as opposed to >30 g/l (63% vs. 4%, respectively).

**Table 1:** Albumin prescription in a clinical scenario

A 75-yr-old woman post-ICU admission day 6, now intubated with pneumosepsis, is requiring a moderate dose of norepinephrine (15 μg/min = 0.2 μg/kg per min) to maintain SBP 90–100 mm Hg with MAP of 55–60 mm Hg. A CXR done earlier today reveals pneumonia but is also consistent with moderate-to-severe pulmonary edema. She now weighs 84 kg, and her preadmission weight was 76 kg. Prehospitalization serum creatinine level was 1.0 mg/dl (92 μmol/l). Laboratory parameters are as follows: creatinine 3.3 mg/dl (292 μmol/l) | urea 19.4 mmol/l | albumin 25 g/l | PaO_2_:FiO_2_ is 240. KRT is started with the aim of fluid removal to improve the patient’s respiratory status.						
“What best describes how likely you would be to prescribe or suggest the administration of albumin during KRT to enhance hemodynamic stability or facilitate fluid removal in this scenario?”						
Responses, *n* (%)	Nephrologists (*n* = 150)		Intensivists (*n* = 53)		Total answered (*N* = 203)	
Very likely	8 (5)		3 (6)		11 (5)	
Likely	25 (17)		9 (17)		34 (17)	
Unlikely	66 (44)		18 (34)		84 (41)	
Very unlikely	51 (34)		23 (43)		74 (37)	
“Regarding this scenario, to what extent would the following changes/additional information make you more or less inclined to prescribe or suggest that albumin be given during KRT to enhance hemodynamic stability or facilitate fluid removal?”						
Responses*, n* (%)		Much less likely to prescribe	Less likely to prescribe	Would not influence my likelihood of prescribing	More likely to prescribe	Much more likely to prescribe
Previous session of KRT in which fluid removal limited by hypotension	Neph.	2 (1)	0	37 (25)	90 (60)	21 (14)
	Intens.	2 (4)	0	24 (45)	21 (40)	6 (11)
	Total	4 (3)	0	61 (30)	111 (55)	27 (12)
KRT modality is CKRT	Neph.	34 (23)	61 (41)	50 (33)	5 (3)	0
	Intens.	5 (9)	11 (21)	35 (66)	2 (4)	0
	Total	39 (19)	72 (35)	85 (41)	7 (3)	0
KRT modality is intermittent hemodialysis	Neph.	1 (0.5)	3 (2)	42 (28)	82 (55)	22 (15)
	Intens.	1 (2)	3(6)	27 (51)	18 (34)	4 (7)
	Total	2 (1)	6 (3)	69 (34)	100 (49)	26 (13)
Requiring high-dose vasopressors	Neph.	4 (2.5)	4 (2.5)	55 (37)	67 (45)	20 (13)
	Intens.	1 (2)	0	17 (32)	26 (49)	9 (17)
	Total	5 (2)	4 (2)	72 (35)	93 (46)	29 (14)
Not requiring vasopressors	Neph.	35 (23)	51 (35)	50 (33)	14 (9)	0
	Intens.	23 (44)	15 (28)	15 (28)	0	0
	Total	58 (29)	66 (32)	65 (32)	14 (7)	0
Serum albumin < 20 g/l	Neph	1 (1)	2 (1)	45 (30)	90 (60)	12 (8)
	Intens.	3 (6)	0	24 (45)	22 (41)	4 (8)
	Total	4 (2)	2 (1)	69 (34)	112 (55)	16 (8)
Serum albumin > 30 g/l	Neph.	42 (28)	45 (30)	58 (39)	5 (3)	0
	Intens.	18 (34)	14 (26)	20 (38)	1 (2)	0
	Total	60 (29)	59 (29)	78 (38)	6 (3)	0
No fluid removal being targeted for this session	Neph.	68 (46)	44 (29)	33 (22)	5 (3)	0
	Intens.	12 (23)	22 (41)	19 (36)	0	0
	Total	80 (39)	66 (32)	52 (26)	5 (2)	0
Extubation planned for after KRT session	Neph.	7 (5)	13 (9)	87 (58)	38 (25)	5 (3)
	Intens.	3 (6)	6 (11)	35 (66)	8 (15)	1 (2)
	Total	10 (5)	19 (9)	122 (60)	46 (23)	6 (3)
If cost of albumin was equal to cost of normal saline	Neph.	2 (1)	5 (3)	102 (68)	33 (22)	8 (5)
	Intens.	3 (6)	1 (2)	33 (62)	14 (26)	2 (4)
	Total	5 (2)	6 (3)	135 (67)	47 (23)	10 (5)

CXR, chest X-ray; CKRT, continuous kidney replacement therapy; FiO_2_, fraction of inspired oxygen; ICU, intensive care unit; Intens., intensivist; KRT, kidney replacement therapy; MAP, mean arterial pressure; Neph., nephrologist; PaO_2_, partial pressure of oxygen; SBP, systolic blood pressure.

As detailed in Supplementary Table S1, although 142 of 214 (66%) agreed that “albumin may facilitate fluid removal,” opinions varied widely regarding the perceived efficacy of i.v. albumin for preventing hypotension, benefiting patients with AKI, its cost-effectiveness, its use as an adjunctive treatment for sepsis, and threshold serum albumin levels for which it should “always” or “never” be prescribed. Most of the respondents (163 of 199 [82%]) agreed that a trial randomizing patient to hyperoncotic albumin versus crystalloid boluses during KRT to evaluate clinically relevant outcomes would be ethical and 146 of 199 (73%) indicated interest in having their institution participate in such a trial.

Our survey of Canadian nephrologists and intensivists indicates that there is a wide self-reported practice variation regarding the use of i.v. albumin in critically ill patients receiving KRT. In addition, our survey suggests that nephrologists and intensivists often use i.v. albumin in this setting (with only 26 of 214 respondents [12%] indicating they “never” do) despite the associated cost and the absence of high-level evidence supporting its use.^7^ Notably, our survey predominantly consisted of physicians practicing at tertiary/academic institutions and may not be representative of community practice.

This survey also identified some potential barriers to implementation of a trial investigating the use of albumin in KRT in ICU, including ensuring buy-in from different stakeholder groups (nephrologists and intensivists) and controlling for the potential impact of the different KRT modalities used across institutions. One of the limitations of this survey is that AKI in the ICU is attributable to multiple causes and, consequently, the use of i.v. albumin may differ based on the etiology of AKI. Nonetheless, general uncertainty regarding the use of i.v. albumin for critically ill patients on KRT, as evidenced by the perception that clinical equipoise exists, could allow for a trial despite wide variation in self-reported current practices. In this context, definitive evidence of either benefit or harm also has great potential to be practice changing.

## Disclosure

SMB reports receiving grants and personal fees from Baxter, BioPorto, I-SPY COVID (National Institutes of Health), and CNA Diagnostics, outside of this manuscript. WBS reports receiving personal fees from Baxter, outside of this manuscript. All the other authors declared no competing interests.

## Supplementary Material

**Supplementary Methods.**

**Table S1.** Albumin survey questions and responses of nephrologists and intensivists.
