# Supplementary material for: The Use of I.V. Albumin During Kidney Replacement Therapy: A Survey of Nephrologists and Intensivists
Source: Kidney Int Rep. 2021 Dec 8;7(3):614–7. doi: 10.1016/j.ekir.2021.11.031 (PMC8897299; doi:10.1016/j.ekir.2021.11.031)
Supplement: Supplementary File (PDF) [file mmc1.pdf]

Supplementary Material

The Use of Intravenous Albumin During Kidney Replacement Therapy: A Survey of Nephrologists and Intensivists

Supplementary Methods- *Page 2*
Supplementary Tables- *Pages 3-11*

Supplementary Methods

An online survey was created by a panel of experts in the use of KRT in critically ill patients (intensivists and nephrologists). It was subsequently reviewed for clarity and face validity by 7 physicians representative of the target population. Invitations with a link to the survey were distributed between September 1st and December 21st, 2020 through Canadian Society of Nephrology (CSN) and Canadian Critical Care Society (CCCS) email distribution lists. After the initial invitation, two subsequent reminders were sent. This study was approved by the Ottawa Health Sciences Network Research Ethics Board (Protocol ID 20190475-01H). The survey included questions that assessed preferences for the use of albumin based on hemodynamics and other clinical parameters in hypothetical patient scenarios. Descriptive statistics were used to summarize the aggregate data with categorical variables using frequencies and percentages. Comparisons of nephrologists' and intensivists' responses were made using the Pearson's χ^2 test with $P < 0.05$ (two-tailed) considered to be statistically significant. The survey questions, including complete reporting of survey responses, are available as Supplementary Material.

SUPPLEMENTARY TABLES

Table S1. Albumin survey questions and responses of nephrologists and intensivists.

**Section A: Demographics**			
	**Nephrologists (N=171)**	**Intensivists (N=59)**	**Total (N=230)**
**1. What is your primary focus of clinical practice?**			
**Adult**	167 (98)	57 (97)	224 (97)
**Pediatric**	4 (2)	2 (3)	6 (3)
**2. What is your specialty training? (Indicate all that are applicable).**			
**Nephrology**	170 (99)	**Critical Care**	57 (97)
**Anesthesia**	5 (3)	**Anesthesia**	11 (19)
**Internal Medicine**	0	**Surgery**	4 (7)
**Other**	48 (28)	**Respirology**	6 (10)
		**Internal medicine**	22 (37)
		**Other**	10 (17)
**3. How many years have you been in clinical practice since obtaining your last specialist qualifications?**			
**I am still in training**	18 (10)	3 (5)	21 (9)
**< 5 years**	20 (12)	15 (25)	35 (15)
**5- 10 years**	27 (16)	13 (22)	40 (17)
**10- 15 years**	34 (20)	10 (17)	44 (19)
**15- 20 years**	34 (20)	3 (5)	37 (16)
**>20 years**	38 (22)	15 (25)	53 (23)
**4. What best describes your primary practice location?**			
**Tertiary/Academic (large [>500 beds] and/or university-affiliated hospital)**	130 (76)	49 (83)	179 (78)
**Metropolitan (moderate size [150-**	32 (19)	10 (17)	42 (18)

**500 beds] community-based hospital)**			
**Regional/rural (smaller [<150 beds] hospital)**	7 (4)	0	7 (3)
**Private hospital**	1 (0.5)	0	1 (0.5)
**Other**	1 (0.5)	0	1 (0.5)
**5. What country do you practice in?**			
**Canada**	159 (93)	54 (92)	213 (93)
**USA**	2 (1)	3 (5)	5 (2)
**Other**	10 (6)	2 (3)	12 (5)
**6. In which settings are you involved in the care of patients receiving renal replacement therapy (RRT) (as hemodialysis and/or SLED and/or CRRT)? (Indicate all that are applicable)**			
**ICU**	163 (95)	57 (97)	220 (96)
**CCU**	121 (71)	5 (8)	126 (55)
**CS-ICU**	84 (49)	18 (31)	102 (44)
**Neuro-ICU**	47 (27)	17 (29)	64 (28)
**None of the above***	8 (5)	0	8 (3)

*Excluded from subsequent questions.

**Section B: Use of albumin for patients receiving RRT in the ICU setting**			
	**Nephrologists (N=162)**	**Intensivists (N=58)**	**Total (N=220)**
**7. In the setting where you are most often involved in the care of critically ill patients receiving RRT, who is responsible for managing the RRT?**			
**Nephrologist only**	93 (58)	3 (5)	96 (44)
**Intensivist only**	4 (2)	16 (28)	20 (9)
**Both nephrologist and intensivist**	61 (38)	37 (64)	98 (44)
**Don’t know**	0	0	0

**Other**	4 (2)	2 (3)	6 (3)
	**Nephrologists (N=157)**	**Intensivists (N=57)**	**Total (N=214)**
**8. What is the RRT modality typically used for critically ill hemodynamically unstable patients at your centre?**			
**CRRT**	109 (69)	45 (79)	154 (72)
**SLED (or PIRRT)**	40 (26)	9 (16)	49 (23)
**Intermittent hemodialysis**	8 (5)	3 (5)	11 (5)
**9. How often do you prescribe, or suggest the use of, albumin fluid to be given to patients receiving RRT (CRRT, SLED of HD) with the goal of improving the hemodynamic tolerance of RRT or facilitating fluid removal?**			
**Always**	0	1 (2)	1 (1)
**Often**	24 (15)	11 (19)	35 (16)
**Occasionally**	51 (33)	22 (39)	73 (34)
**Rarely**	65 (41)	14 (24)	79 (37)
**Never**	17 (11)	9 (16)	26 (12)

**10. On a scale of 1 to 5, specify to what extent to which you agree with the following statements regarding the administration of albumin fluid during RRT?**						
		**Strongly Disagree**	**Disagree**	**Neither Agree Nor Disagree**	**Agree**	**Strongly Agree**
**Albumin can prevent hypotension during dialysis**	**Nephrologists (N=157)**	6 (4)	22 (14)	54 (34)	68 (43)	7 (5)
	**Intensivists (N=57)**	1 (2)	15 (26)	23 (40)	15 (26)	3 (5)
	**Total answered (N=214)**	7 (3)	37 (17)	77 (36)	83 (39)	10 (5)
**If fluid is required to treat**	**Nephrologists (N=157)**	10 (6)	45 (29)	56 (36)	40 (25)	6 (4)

**hypotension during dialysis, albumin is more effective than crystalloids**	**Intensivists (N=57)**	4 (7)	13 (23)	20 (35)	17 (30)	3 (5)
	**Total (N=214)**	14 (7)	58 (27)	76 (35)	57 (27)	9 (4)
**Hypoalbuminemia is harmful**	**Nephrologists (N=157)**	9 (6)	36 (23)	46 (29)	57 (36)	9 (6)
	**Intensivists (N=57)**	8 (14)	20 (35)	18 (32)	10 (17)	1 (2)
	**Total (N=214)**	17 (8)	56 (26)	64 (30)	67 (31)	10 (5)
**Albumin may especially benefit patients with acute kidney injury**	**Nephrologists (N=157)**	16 (10)	66 (42)	59 (38)	16 (10)	0
	**Intensivists (N=57)**	6 (11)	25 (44)	23 (40)	3 (5)	0
	**Total (N=214)**	22 (10)	91 (42)	82 (38)	19 (9)	0
**Albumin may especially benefit patients with end-stage kidney disease**	**Nephrologists (N=157)**	27 (17)	64 (41)	58 (37)	8 (5)	0
	**Intensivists (N=57)**	9 (16)	25 (44)	21 (37)	2 (3)	0
	**Total (N=214)**	36 (17)	89 (42)	79 (37)	10 (5)	0
**Albumin may facilitate fluid removal**	**Nephrologists (N=157)**	4 (2)	11 (7)	34 (22)	96 (61)	12 (8)
	**Intensivists (N=57)**	3 (5)	9 (16)	11 (19)	31 (55)	3 (5)
	**Total (N=214)**	7 (3)	20 (9)	45 (21)	127 (59)	15 (7)
**Albumin is an adjunctive treatment for sepsis**	**Nephrologists (N=157)**	31 (20)	66 (42)	44 (28)	15 (9)	1 (1)
	**Intensivists (N=57)**	17 (30)	19 (33)	10 (18)	9 (16)	2 (3)

	**Total (N=214)**	48 (22)	85 (40)	54 (25)	24 (11)	3 (1)
**Albumin is too expensive for routine use in RRT patients**	**Nephrologists (N=157)**	7 (4)	31 (20)	38 (24)	52 (33)	29 (18)
	**Intensivists (N=57)**	3 (5)	12 (21)	12 (21)	17 (30)	13 (23)
	**Total (N=214)**	10 (5)	43 (20)	50 (23)	69 (32)	42 (20)
**Albumin is less likely to be beneficial for patients on CRRT vs. intermittent HD**	**Nephrologists (N=157)**	2 (1)	30 (19)	49 (31)	69 (44)	7 (4)
	**Intensivists (N=57)**	2 (3)	15 (26)	29 (51)	10 (18)	1 (2)
	**Total (N=214)**	4 (2)	45 (21)	78 (36)	79 (70)	8 (4)

	**Nephrologists (N=81)**	**Intensivists (N=26)**
**11. Are there other factors that you consider when deciding whether or not to prescribe albumin for patients while they are receiving RRT? (most common responses from respondents)**		
**Liver cirrhosis**	12 (7)	4 (7)
**Fluid removal**	6 (3.5)	2 (3)
**Serum albumin level**	8 (5)	4 (7)
**Staff comfort to prescribe albumin**	1 (0.5)	1 (1.5)
**Severity of hypoalbuminemia**	11 (6)	2 (3)
**Risk of blood product**	2 (1)	1 (1.5)
**Presence of edema**	4 (2)	1 (1.5)
**If patient is on vasopressor**	1 (0.5)	2 (3)

**Personal preference of not prescribing albumin**	3 (1.5)	2 (3)	
**No evidence that albumin works**	3 (1.5)	-	
**When nothing else works**	4 (2)	-	
**12. Do you have a threshold serum albumin concentration over which you would NEVER prescribe (or suggest the use of), albumin to be given during RRT?**			
	**Nephrologists (N=157)**	**Intensivists (N=57)**	**Total (N=214)**
**I don’t have a threshold for this**	60 (38)	25 (44)	85 (35)
**>=40 g/L**	9 (6)	1 (2)	10 (5)
**>=35 g/L**	26 (17)	10 (17)	36 (17)
**>=30 g/L**	40 (25)	14 (25)	54 (25)
**>=25 g/L**	10 (6)	2 (3)	12 (5.5)
**>=20 g/L**	8 (5)	5 (9)	13 (6)
**>=15 g/L**	1 (0.5)	0	1 (0.5)
**Other**	3 (2.5)	0	3 (1)
**13. Do you have a threshold serum albumin concentration over which you would ALWAYS prescribe (or suggest the use of), albumin to be given during RRT?**			
**I don’t have a threshold for this**	137 (87)	50 (88)	187 (87)
**>=40 g/L**	0	0	0
**>=35 g/L**	1 (0.5)	0	1 (0.5)
**>=30 g/L**	4 (2.5)	0	4 (2)
**>=25 g/L**	3 (2)	2 (4)	5 (2.5)
**>=20 g/L**	7 (5)	3 (5)	10 (5)
**>=15 g/L**	3 (2)	2 (3)	5 (2.5)

**Other**	2 (1)	0	2 (1)

Section C: Clinical scenario
This section is reported in Table 1 in manuscript Chan *et al*, 2021.

A 75-year-old woman post-ICU admission day 6, now intubated with pneumosepsis, is requiring a moderate dose of norepinephrine (15 mcg/min = 0.2 mcg/kg/min) to maintain SBP 90 to 100 mmHg with MAP of 55 to 60 mmHg. A CXR done earlier today shows pneumonia but is also consistent with moderate to severe pulmonary edema.
She now weighs 84 kg and her pre-admission weight was 76 kg. Pre-hospitalization serum creatinine was 1.0 mg/dL (92 umol/L)
Laboratory parameters are as follows: Creatinine 3.3 mg/dL (292 umol/L) | urea 19.4 mmol/L | albumin 25 g/L | PaO2:FiO2 is 240
KRT is started with the aim of fluid removal to improve the patient's respiratory status.

"What best describes how likely you would be to prescribe or suggest the administration of albumin during KRT in order to enhance hemodynamic stability or facilitate fluid removal in this scenario?"

**Responses, N (%)**	**Nephrologists (N=150)**	**Intensivists (N=53)**	**Total answered (N=203)**
Very likely	8 (5)	3 (6)	11 (5)
Likely	25 (17)	9 (17)	34 (17)
Unlikely	66 (44)	18 (34)	84 (41)
Very unlikely	51 (34)	23 (43)	74 (37)

"Regarding this scenario, to what extent would the following changes/additional information make you more or less inclined to prescribe or suggest that albumin be given during KRT in order to enhance hemodynamic stability or facilitate fluid removal?"

**Responses, N (%)**		**Much less likely to prescribe**	**Less likely to prescribe**	**Would not influence my likelihood of prescribing**	**More likely to prescribe**	**Much more likely to prescribe**
Previous session of KRT in which fluid removal limited by hypotension	Neph.	2 (1)	0	37 (25)	90 (60)	21 (14)
	Intens.	2 (4)	0	24 (45)	21 (40)	6 (11)
	Total	4 (3)	0	61 (30)	111 (55)	27 (12)
KRT modality is CKRT	Neph.	34 (23)	61 (41)	50 (33)	5 (3)	0
	Intens.	5 (9)	11 (21)	35 (66)	2 (4)	0

	Total	39 (19)	72 (35)	85 (41)	7 (3)	0
KRT modality is intermittent hemodialysis	Neph.	1 (0.5)	3 (2)	42 (28)	82 (55)	22 (15)
	Intens.	1 (2)	3(6)	27 (51)	18 (34)	4 (7)
	Total	2 (1)	6 (3)	69 (34)	100 (49)	26 (13)
Requiring high-dose vasopressors	Neph.	4 (2.5)	4 (2.5)	55 (37)	67 (45)	20 (13)
	Intens.	1 (2)	0	17 (32)	26 (49)	9 (17)
	Total	5 (2)	4 (2)	72 (35)	93 (46)	29 (14)
Not requiring vasopressors	Neph.	35 (23)	51 (35)	50 (33)	14 (9)	0
	Intens.	23 (44)	15 (28)	15 (28)	0	0
	Total	58 (29)	66 (32)	65 (32)	14 (7)	0
Serum albumin <20 g/L	Neph	1 (1)	2 (1)	45 (30)	90 (60)	12 (8)
	Intens.	3 (6)	0	24 (45)	22 (41)	4 (8)
	Total	4 (2)	2 (1)	69 (34)	112 (55)	16 (8)
Serum albumin >30 g/L	Neph.	42 (28)	45 (30)	58 (39)	5 (3)	0
	Intens.	18 (34)	14 (26)	20 (38)	1 (2)	0
	Total	60 (29)	59 (29)	78 (38)	6 (3)	0
No fluid removal being targeted for this session	Neph.	68 (46)	44 (29)	33 (22)	5 (3)	0
	Intens.	12 (23)	22 (41)	19 (36)	0	0
	Total	80 (39)	66 (32)	52 (26)	5 (2)	0
Extubation planned for after KRT session	Neph.	7 (5)	13 (9)	87 (58)	38 (25)	5 (3)
	Intens.	3 (6)	6 (11)	35 (66)	8 (15)	1 (2)
	Total	10 (5)	19 (9)	122 (60)	46 (23)	6 (3)
If cost of albumin was equal to cost of normal saline	Neph.	2 (1)	5 (3)	102 (68)	33 (22)	8 (5)
	Intens.	3 (6)	1 (2)	33 (62)	14 (26)	2 (4)
	Total	5 (2)	6 (3)	135 (67)	47 (23)	10 (5)

Section D: Interest in participating in future clinical trials assessing the use of albumin in patients receiving KRT.			
**13. Volume overload and hemodynamic instability during RRT may both negatively impact kidney recovery in critically ill patients with AKI. Administration of intravenous albumin during RRT might potentially help mitigate against hemodynamic instability and facilitate fluid removal but is expensive and does come with some theoretical risks. For critically ill hypoalbuminemic patients with AKI, would you consider it ethical to conduct a trial that randomized patients to 25% albumin vs normal saline boluses during RRT in order to assess its impact on outcomes such as 'time to kidney recovery/dialysis-independence' and 'duration of mechanical ventilation'?**			
	**Nephrologists (N=147)**	**Intensivists (N=52)**	**Total (N=199)**
**Yes**	122 (83)	41 (79)	163 (82)
**No**	8 (5)	3 (5)	11 (6)
**Unsure**	17 (12)	4 (8)	21 (10)
**Other**	0	4 (8)	4 (2)
**14. Would you be interested in having your centre participate in such a trial?**			
**Yes**	107 (73)	39 (75)	146 (73)
**No**	27 (18)	8 (15)	35 (18)
**Other**	13 (9)	5 (10)	18 (9)
